# Analysis of Mechanical Properties and Thermal Conductivity of Thin-Ply Laminates in Ambient and Cryogenic Conditions

**DOI:** 10.3390/ma17225419

**Published:** 2024-11-06

**Authors:** Anna Krzak, Agnieszka J. Nowak, Jiří Frolec, Tomáš Králík, Maciej Kotyk, Dariusz Boroński, Grzegorz Matula

**Affiliations:** 1 Scientific and Didactic Laboratory of Nanotechnology and Material Technologies, Silesian University of Technology, 44-100 Gliwice, Poland; agnieszka.j.nowak@polsl.pl (A.J.N.); grzegorz.matula@polsl.pl (G.M.); 2 Institute of Scientific Instruments, Czech Academy of Sciences, Kralovopolska 147, 612 00 Brno, Czech Republic; frolec@isibrno.cz (J.F.); kralik@isibrno.cz (T.K.); 3 Faculty of Mechanical Engineering, Bydgoszcz University of Science and Technology, Al. Prof. S. Kaliskiego 7, 85-796 Bydgoszcz, Poland; maciej.kotyk@pbs.edu.pl (M.K.); dariusz.boronski@pbs.edu.pl (D.B.)

**Keywords:** mechanical testing, thermal testing, composite, cryogenic, epoxy–glass laminate

## Abstract

It is widely known that glass–epoxy laminates are renowned for their high stiffness, good thermal properties, and economic qualities. For this reason, composite materials find successful applications in various industrial sectors such as aerospace, astronautics, the storage sector, and energy. The aim of this study was to investigate the mechanical and thermal properties of composite materials comprising two different types of epoxy resin and three different hardeners, both at room temperature and under cryogenic conditions. The samples were produced at IZOERG (Gliwice, Poland) using a laboratory hot-hydraulic-press technique. During cyclic loading–unloading tests, degradation up to a strain level of 0.6% was observed both at room temperature (RT) and at 77 K. For a glass-reinforced composite with YDPN resin (EP_1_1), the highest degradation was recorded at 18.84% at RT and 33.63% at 77 K. We have also investigated the temperature dependence of thermal conductivity for all samples in a wide temperature range down to 5 K. The thermal conductivity was found to be low and had a relative difference of up to 20% among the composites. The experimental results indicated that composites under cryogenic conditions exhibited less damage and were stiffer. It was confirmed that the choice of hardener significantly influenced both properties.

## 1. Introduction

Composite materials reinforced with glass fabric (GFRP) are classified among the most complex and advanced groups of engineering materials. They are widely used and highly regarded in cryogenic engineering applications and the aerospace industry due to their exceptional properties, such as their high specific strength and stiffness, excellent fatigue resistance, and high corrosion resistance [1,2]. On the other hand, the most commonly used matrices include epoxy resins, which in recent years have played an important role in the field of polymer materials due to their exceptional thermal and mechanical properties [3]. They are widely used as resin matrices in composite materials [4,5,6], cryogenic adhesives [7], insulating materials [8], in coils [9], and in superconducting magnets [3,10]. Advancing aviation technology requires epoxy materials to perform reliably at both elevated and cryogenic temperatures for space exploration. Currently, work is underway on degradable epoxy resins that are suitable for recycling in closed-loop processes [11]. The scientific literature contains extensive information on the impact of cryogenic conditions on the mechanical and thermal properties of composite materials [12,13,14], but there is still a lack of precise comparisons between experimental results obtained on a laboratory and industrial scale. Most of the literature reports primarily on modifying the resin itself, as the matrix material, to increase its ductility under extreme temperature conditions [12,15]. The rest focus on introducing a specific reinforcing phase in the form of particles or fibers to minimize the effects of low temperatures on the entire composite—limiting the initiation and propagation of microcracks [13,16]. Currently, the studies described in the literature concerning materials used at cryogenic temperatures are difficult to compare and undoubtedly require organization. The main problem associated with the use of these materials at low temperatures is their negative impact on the following properties: ductility, deformability, and impact strength. The drop in temperature to cryogenic levels, from 223 K to at least 123 K, causes the formation of internal stresses in the polymer matrix due to thermal contraction. As a result of this phenomenon, when the stress intensity factor induced by thermal stress exceeds the resin′s fracture resistance, microcracks appear in the composite, which are dangerous from an operational standpoint. The fracture resistance of the matrix at cryogenic temperatures can be improved by controlling the structure of the applied chemical substance (network structure and morphology control) [12]. Consequently, the microstructure becomes more orderly at such low temperatures. Ghosh [17] examined the tensile properties of the composite material for use as the neck of a cryogenic vessel. The results show that for composites conditioned at 77 K in a liquid nitrogen bath for 15 min, 30 min, 45 min, and 60 min, the strength and modulus significantly increase with the increase in strain. Previous studies, which also form the continuation of this work [18,19,20,21], have shown that cryogenic temperatures contribute to the improvement in mechanical properties (impact strength, bending, and tensile properties). Among the best materials are composite materials that use brominated epoxy resin, DICY hardener, and 70/30 anhydride. It is also worth considering that, recently, thin-ply materials have been gaining increasing popularity due to their greater design possibilities [22]. The first benefit is the ability to use a greater number of layer orientations to achieve an optimal solution, and the second is the lower production costs [22,23]. All objects used by The National Aeronautics and Space Administration (NASA) and The European Space Agency (ESA) need to be as light as possible. Projects often identify engineering materials such as alloys, polymers, and composites as candidates to meet these requirements, based on their known properties at room temperature. As a result, NASA often requires data on the thermal conductivity of materials at cryogenic temperatures [24]. In the scientific literature, numerous experimental studies on the thermal conductivity of FRP composites can be found, with some focusing on thermal conductivity in cryogenic environments [25,26,27,28]. The thermal conductivity of a material often depends on temperature, composition, impurities, structural defects, and other factors. In the article by Amils [29], it is noted that applications such as astrophysics and deep space communication require state-of-the-art performance with low noise levels. To function correctly, these devices need good electrical contact between the back of the integrated circuit and the housing, and, ideally, a low thermal conductivity of the epoxy resin compared to GaAs. The low thermal conductivity of the epoxy resin ensures that an acceptable temperature rise can be achieved at the microwave termination and sensor without dissipating a large amount of power [30].

In this paper, a research direction was chosen that combines two aspects: parallel work on resin modification and the development of a spatial reinforcing phase. The project aims to develop an advanced composite material with a thermosetting matrix reinforced by a structural reinforcing phase. The impact of the applied component modifications on the mechanical and thermal properties of the finished composite material was assessed at both cryogenic and room temperatures to obtain a full spectrum of the material’s behavior across a complex temperature range.

## 2. Experiments

### Materials

For this study, the GFRP composite samples were fabricated by a hot-press process using E glass fabric (205 g/m^2^) and epoxy resin with different types of hardeners, as shown in Table 1. The glass fiber and resin weight content were optimized from 70% to 80% and 30% to 35%. The epoxy resin (EP), the specific type of epoxy resin (X), and the type of hardener (Y) were used to identify the composite materials. The designation format was defined as EP_X_Y.

References [18,19,20,21] contain detailed information on the production of composite sheets. The epoxy–glass composite production process involves coating raw glass fabric with epoxy resin, followed by heat treatment in a drying tunnel to evaporate and catalytically burn the solvent. After achieving the necessary resin condensation, the dried fabric is layered and pressed in a hydraulic press under pressure (10 MPa) and elevated temperature (433 K).

## 3. Measurement Characterization

### 3.1. Thermal Conductivity

**Figure 1:**
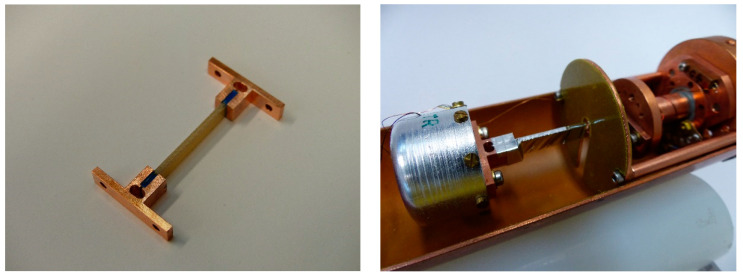
An example of the bare sample fixed between the copper flanges for thermal conductivity testing (**left**) and its configuration inside the measuring chamber after the application of the shielding aluminized Mylar foil (**right**).

A dedicated apparatus for the measurement of the thermal conductivity of metallic samples at low temperatures (Institute of Scientific Instruments of the CAS, Brno, Czech Republic [31]) has been modified and used for the measurement of thermal conductivity in this study (Figure 1, right). The method is based on steady-state calorimetry, by using a built-in heat flow meter made of a thermal resistor and two thermometers at its ends. The first modification resides in the bonding of the composite sample ends with epoxy LOCTITE® Stycast 2850 FT™ with catalyst CAT 23 LV™ (Henkel AG & Co. KGaA, Düsseldorf, Germany) to copper flanges (Figure 1, left). The epoxy is hardened at room temperature and then the sample is installed into the measurement chamber. In order to minimize the parasitic leak of the thermal radiation from the hot end (at higher *T_R_*), the sample is wrapped around by a strip of double-aluminized Mylar® foil (Beyond Gravity GmbH, Vienna, Austria) of 5 mm width. Wrapping is carried out spirally, in order to minimize heat conduction via the foil. The sample is placed in a cylindrical measuring chamber, which is inserted into a stainless-steel casing tube. The tube is evacuated and immersed in a Dewar vessel (Cryotherm GmbH & Co. KG, Kirchen, Germany) with liquid helium. The temperatures *T_R_* of the radiator (hot end of the sample), absorber (*T_A_* at the cold end of the sample), and bottom of the heat-flow meter (*T_B_*) are monitored during the measurement using temperature controller Lake Shore 340 (Lake Shore Cryotronics, Inc., Westerville, United States) The radiator temperature *T_R_* is set to selected setpoints (from 5 K to 300 K) and after reaching thermal equilibrium, the temperatures *T_R_*, *T_A_*, and *T_B_* are read. The apparatus measures the integral thermal conductivity κ(*T_A_*, *T_R_*).

(1) $$\kappa \left(T_{A},T_{R}\right)\equiv \int_{T_{A}}^{T_{R}}\lambda \left(T\right)dT=Q\frac{A}{L},$$

where *λ* is the specific thermal conductivity of the sample, *Q* represents the heat power transferred via the sample, *A* is its cross-section area, and *L* is the length of the sample between the thermal contacts. The specific thermal conductivity *λ(T)* is evaluated by the derivation (2) of *κ* at selected setpoints.

(2) $$\frac{d}{dT_{R}}\kappa \left(T_{R},T_{A}\right)=\lambda \left(T_{R}\right)-\lambda \left(T_{A}\right)\frac{dT_{A}}{dT_{R}}$$

Thermal conductivity *λ(T)* can be calculated by the derivation of *κ*(*T_A_*, *T_R_*) with respect to *T_R_*, when temperature *T_A_* remains constant. This condition is not exactly met in our experiment configurations. However, the *T_A_* values rise only fractionally with an increasing *T_R_* due to the temperature gradient on the thermal resistor, which ranges from approximately 4.3 K to 5.7 K (for T_R_ increasing from 4.5 K to 300 K). The relative value of the second term in Equation (2) is between 0.2% and 0.5% in the whole *T_R_* temperature range for low-conductivity composite samples.

### 3.2. Loading/Unloading Cyclic Tensile Tests

**Figure 2:**
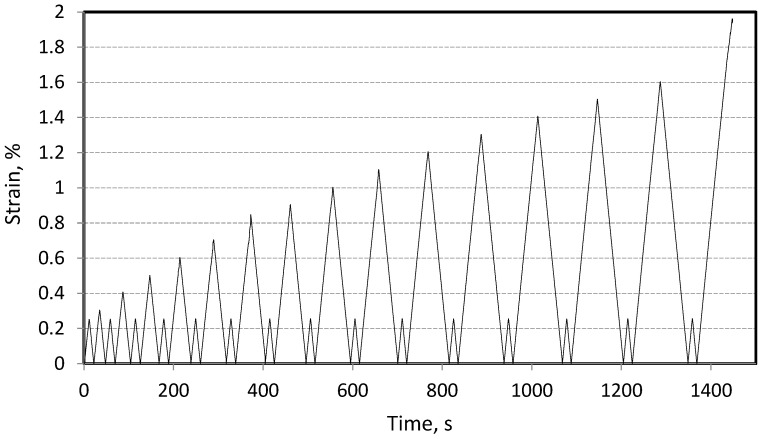
Illustration of load application in loading–unloading tests.

**Figure 3:**
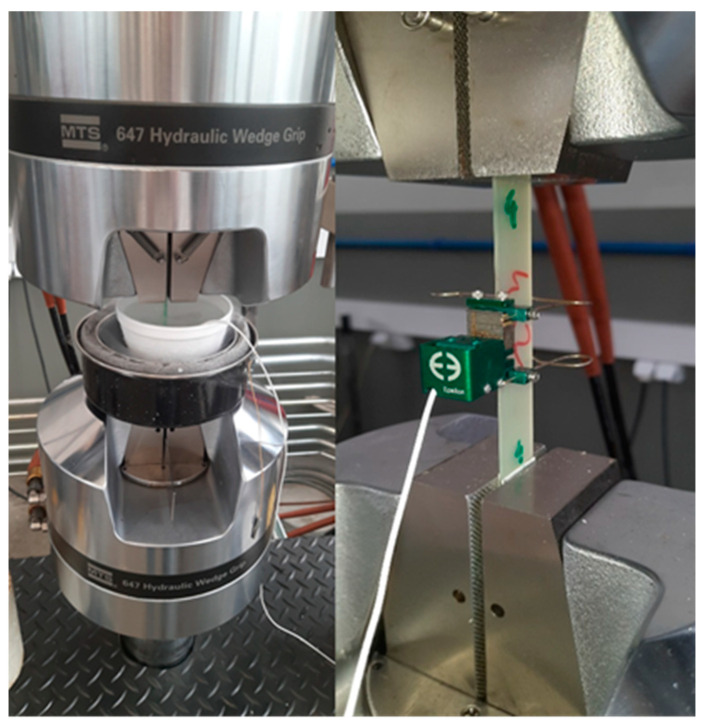
An example of the loading–unloading tests: the clamping of the sample (**right**) and measurement in LN_2_ (**left**).

Tensile tests were performed using an Instron 8502 ServoHydraulic Fatigue Testing Systems equipped with a 250 kN load cell and hydraulic grips. Axial strains were measured using a standard dynamic Epsilon 3442-LT Axial Extensometer (Epsilon Technology Corporation, Logan, UT, USA) with a 20 mm base, positioned in the middle of the sample’s gauge length. Due to practical limitations, only three specimens for each test were used to obtain the presented results. Undesired loads resulting from the contraction of the specimens and the equipment parts during chamber cooling were compensated until thermal equilibrium was reached. The tests were conducted in the displacement control mode at a rate of 2 mm/min. The modulus was determined from stress–strain curves within a linear range from 0.005% to 0.002% strain. In the cyclic loading–unloading tests (L–UL), the samples underwent steps of increasing strain, starting from 0.25% until failure (or up to a maximum strain of 1.60%). After each loading step, the samples were unloaded to 15 N, the axial strains were zeroed, and a low-level (up to 0.25%) L–UL step was performed, from which the modulus was determined. The tests were conducted in accordance with ASTM D3039 standards [32]. Figure 2 shows the schematic representation of the load progression up to 1.6%, followed by an increase in load until the sample failure. Figure 3 shows the clamping of the samples along with the extensometer used.

## 4. Results and Discussion

### 4.1. Thermal Properties

**Figure 4:**
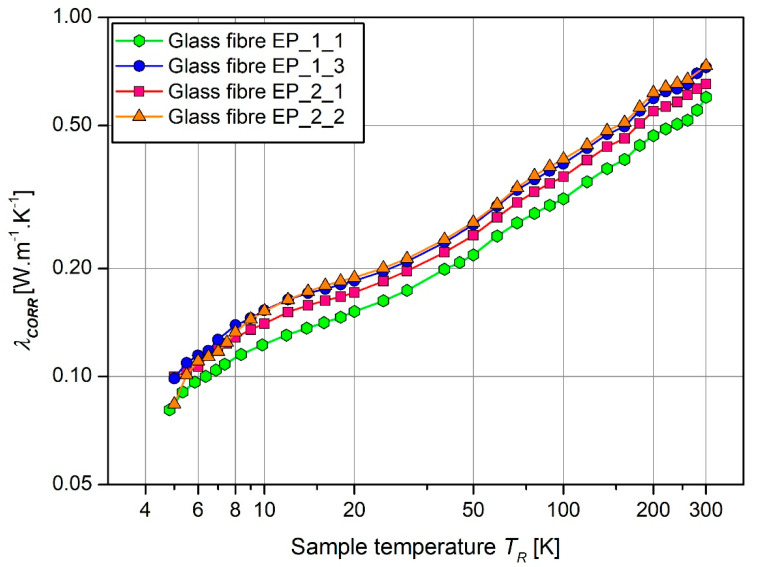
Thermal conductivity of epoxy laminates in range of 5 K to 300 K.

Figure 4 and Table 2 present the obtained results of the thermal conductivity of composite materials in the temperature range from 5 K to 300 K.

As can be seen from Figure 4, the thermal conductivity raises with increasing temperature. Based on Table 2, it is noticeable that composite materials using Novolak P as a hardener exhibit lower thermal conductivity values. A slightly higher thermal conductivity of around 0.7 [W/m/K] was observed for samples with DDS and DICY hardeners. The difference between the various extreme results is approximately 23%. Compared to the literature [33], it is confirmed that the reinforcing phase has a more significant impact, and the epoxy composition can be modified with agents that improve thermal conductivity. These include carbon-based fillers, carbon nanotubes, soot, graphene, and metallic, ceramic, and plant-based fillers [34,35]. Hybrid fillers are also being developed [36,37]. For comparison, composites used in the field of aviation and astronautics had thermal conductivities of 1.5 W/m/K [38], 0.84 W/m/K [39], and 0.35 W/m/K [40].

### 4.2. Mechanical Properties

**Figure 5:**
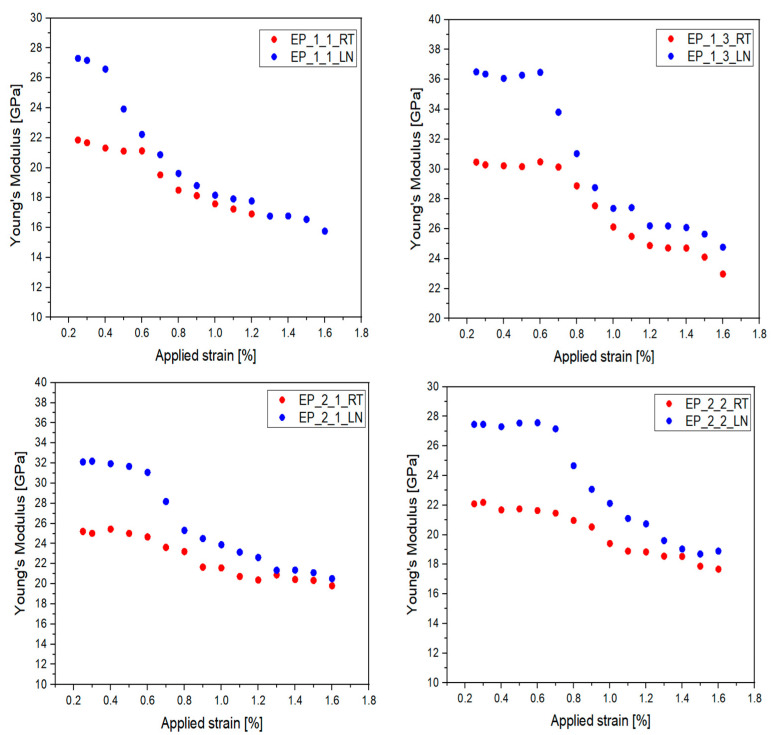
Degradation of Young’s modulus in epoxy laminates with increasing applied strain at room temperature (RT) and at boiling point of liquid nitrogen (LN_2_; 77 K) (RT, LN_2_; 77 K).

The purpose of the cyclic tensile tests, conducted at room temperature (RT) and in a low-temperature environment (liquid nitrogen—LN_2_) at 77 K (Figure 5, was to understand the material properties under critical mechanical conditions. The choice of the testing temperatures resides in the fact that LN_2_ is one of the most widely used cryo-liquids, while the outer parts of cryogenic systems remain at room temperature. Table 3 summarizes the strength values of the laminates for all tested composites at room temperature and 77 K. For easier comparison, Table 4 provides the reduced Young’s modulus values at 1.1% strain for all the tested composites at room temperature and 77 K. Figure 5 illustrates the degradation of materials, represented by the reduction in Young’s modulus, as the applied strain increased at room temperature and 77 K.

Experimental studies confirm a fact mentioned in all the scientific literature—a low temperature significantly increases the stiffness of composite materials. Analyzing Figure 4, it can be observed that a low temperature leads to a significant or high difference in Young′s modulus compared to room temperature. The EP_1_1 material degrades at a strain level of 0.6% at room temperature (RT), while it fails at 0.4% in liquid nitrogen (LN_2_), making it unsuitable for cryogenic applications. The sample becomes too brittle. In terms of maximum strain, stress at both RT and in LN_2_, and degradation, the most promising composite material, although requiring composition modifications, is the EP_1_3 sample.

Comparing the above-described materials, which have the same type of resin (YDPN) but different hardeners (Novolac P and DDS), it can be observed that the degradation of Young′s modulus at RT is similar. However, in LN_2_, the situation is different—for EP_1_1, it is around 34%, whereas for EP_1_3, the degradation is slightly lower at around 25%. Materials using the YD-128 epoxy resin exhibit poorer mechanical properties—the Young′s modulus of both materials at RT is below 25 GPa. In cryogenic conditions, it is close to 30 GPa, which results in a significant disparity between RT and LN_2_.

When comparing these results with previous studies [18,19] conducted at ambient (RT) and 223 K (Table 5), it is crucial to take into account several factors that significantly influence the outcomes, such as the production batch, the sample cutting location, the laboratory coater (which has a higher margin of error and lacks repeatability), and the testing equipment. In the studies discussed, the composite materials were produced on a laboratory scale to avoid costs and time limitations. However, there are plans to implement selected composite materials on a production scale in the future.

The primary goal of this work was to compare the mechanical properties of the experimental results measured at 77 K with the data from the previous scientific study [18,19]. Table 5 presents the comparative data of the tested materials at 223 K. Interpreting the results from Table 5 and Figure 5, the following observations can be made: the materials from previous studies exhibit better performance compared to the current experimental results, indicating that they are more stable at both RT and 223 K. This supports the analysis of the EP_1_1 and EP_1_3 materials—the former shows poorer properties and will be excluded from consideration for cryogenic applications. Conversely, the results for EP_1_3 confirm the consistency of the findings.

Adding woven material increases the laminate′s stiffness, reducing deformations during the automated placement of thermo-plastic composites [41]. It presents a future option for the use of hybrid materials.

However, these results highlight that composite materials belong to a complex engineering category. Reproducing identical laminates from different production batches and laboratory compositions is not feasible, as production factors always influence the outcome. In the future, industrial-scale production is planned, and it would be beneficial to conduct a full mechanical and thermal characterization on the same material from the same production batch.

Additionally, to expand our knowledge, the samples were exposed to liquid helium (LHe). Figures S5–S8 present microphotographs of the surface of composite materials 500× magnification. These microphotographs were taken using the FIB-SEM Helios scanning electron microscope equipped with an Everhart-Thornley detector (Thermo Fisher Scientific, Waltham, MA, USA). The images were taken at a 1 keV primary electron energy and 50 pA current. They were pictured without coating using the Secondary electrons mode. Detected secondary electrons mainly show the topography of the sample.

Based on these observations, it appears that the treatment in LHe had no effect. Several imperfections were observed, such as indentations, undissolved epoxy resin, and some mechanical damage likely caused during manufacturing or cutting. No microcracks resulting from cryogenic exposure were detected.

The Supplementary Materials include additional SEM images at various magnifications and images captured with a Leica DVM6 digital microscope, capable of precise 2D and 3D image analysis at 30× magnification.

## 5. Conclusions

In this article, the impact of various matrix modifications on the mechanical and thermal properties of E–glass fiber-reinforced epoxy resin composites is examined in detail. The results presented in this paper show that the type of hardener and epoxy resin used has a significant effect on the behavior of the materials both at room temperature and under cryogenic conditions.

The primary findings are as follows.

- Mechanical and thermal properties are closely linked—in the case of EP_1_1, the lowest values were recorded in both properties. The Young′s modulus at room temperature (RT) was 21.71, and at liquid nitrogen temperature (LN_2_) it was 27.30. Additionally, the degradation of the Young′s modulus at RT occurs at a level of 0.6%, and at LN_2_ it is 0.4%.
- The highest Young′s Modulus values at room temperature (30 GPa) and in liquid nitrogen (37 GPa) were recorded for EP_1_3. This material consists of YPDN epoxy resin and DDS. The same applies to maximum stress—both at room temperature and in liquid nitrogen, it is around 400 MPa.
- The thermal conductivity of EP_1_1 at RT is 0.6 W/m/K, and at LN_2_ it is 0.3 W/m/K. Values of thermal conductivity in the case of the other tested composites are slightly higher. As a result of the study, it was found that the material requires further work on modifying the resin composition to improve both mechanical and thermal properties.
- SEM did not detect any microcracking caused by cryogenic LHe exposure.

In the future, numerical simulations are planned to characterize the mechanical performance of composite materials for potential use as cryogenic tanks. The next step will also involve increasing the cryogenic cycles in LHe to verify mechanical properties and detect any microcracks induced by thermal shock.

Although EP_1_1 has inferior properties compared to the other composite materials tested, it may have excellent potential for application in a different field. The samples have been sent for further testing to develop a complete material profile, necessary for future market implementation.

## Figures and Tables

**Table 1:** The designation of composite materials.

Symbol	Resin Type	Hardener	Epoxy Manufacturer
EP_1_1	YDPN	Novolac P	Kukdo (Seoul, Republic of Korea)
EP_1_3	YDPN	DDS	
EP_2_1	YD-128	Novolac	Aditya Birla Chemical (Rayong, Thailand)
EP_2_2	YD-128	DICY	

**Table 2:** A comparison of the thermal conductivity values measured on the presented samples.

Material	Thermal Conductivity [W/m/K] at 300 K	Thermal Conductivity [W/m/K] at 77 K	Thermal Conductivity [W/m/K] at 5 K
EP_1_1	0.60	0.29	0.08
EP_1_3	0.73	0.35	0.10
EP_2_1	0.65	0.33	0.10
EP_2_2	0.73	0.36	0.08

**Table 3:** Maximum stress and strain of composite laminates at room temperature (RT) and in liquid nitrogen (LN_2_; 77 K).

Material	Max Stress at RT [MPa]	Max Stress at LN_2_ [MPa]	Max Strain at RT [%]	Max Strain at LN_2_ [%]
EP_1_1	221.94	287.81	1.44	1.87
EP_1_3	398.54	382.47	2.00	1.63
EP_2_1	341.64	365.17	1.84	1.98
EP_2_2	324.51	301.62	2.1	1.79

Considering that the grips were not submerged in liquid nitrogen, cracks may have occurred in the higher temperature zone of the sample. Therefore, the maximum stress values determined for samples tested in liquid nitrogen represent the minimum strength of the sample after cyclic loading.

**Table 4:** Young’s modulus *E* of composite laminates in different conditions (RT or LN_2_) at 1.1%.

Material	Initial *E* at RT [GPa]	*E* at 1.1% at RT [GPa]	Δ*E_RT_* [%]	Initial *E* at LN_2_ [GPa]	*E* at 1.1% at LN_2_ [GPa]	Δ*E _LN2_* [%]
EP_1_1	21.71	17.62	18.84	27.30	18.12	33.63
EP_1_3	30.35	25.58	15.72	37.45	27.94	25.40
EP_2_1	24.98	20.62	17.45	31.69	22.79	28.10
EP_2_2	22.00	19.22	12.64	27.58	20.90	24.22

**Table 5:** Young’s modulus *E* of composite laminates in different conditions at 1.1% from other research [18,19].

Material	Initial *E* at RT [GPa]	*E* at 1.1% at RT [GPa]	Δ*E_RT_* [%]	Initial *E* at 223 K [GPa]	*E* at 1.1% at 223 K [GPa]	Δ*E_223 K_* [%]
EP_1_1	27.4	24.5	10.6	29.8	24.8	16.8
EP_1_3	35.6	32.8	7.9	37.8	34.3	9.3
EP_2_1	29.8	27.3	8.4	30.9	28.4	8.1
EP_2_2	27.9	26.3	5.7	30.6	28.4	7.2

## Data Availability

The raw data supporting the conclusions of this article will be made available by the authors on request.

## Supplementary Materials

The following supporting information can be downloaded at: https://www.mdpi.com/article/10.3390/ma17225419/s1, Figure S1: Microscope images of EP_1_1 laminates in 30× magnification, tested in RT (left) and in LN (right); Figure S2: Microscope images of EP_1_3 laminates in 30× magnification, tested in RT (left) and in LN (right); Figure S3: Microscope images of EP_2_1 laminates in 30× magnification, tested in RT (left) and in LN (right); Figure S4: Microscope images of EP_2_2 laminates in 30× magnification, tested in RT (left) and in LN (right); Figure S5: SEM image of EP_1_1 laminate at 500× magnification taken at RT before (left) and after (right) immersion of the sample in LHe; Figure S6: SEM image of EP_1_3 laminate at 500× magnification, taken at RT before (left) and after (right) immersion of the sample in LHe; Figure S7: SEM image of EP_2_1 laminate at 500× magnification, taken at RT before (left) and after (right) immersion of the sample in LHe; Figure S8: SEM image of EP_2_2 laminate at 500× magnification, taken at RT before (left) and after (right) immersion of the sample in LHe.
