# Supplementary material for: Analysis of Mechanical Properties and Thermal Conductivity of Thin-Ply Laminates in Ambient and Cryogenic Conditions
Source: Materials (Basel). 2024 Nov 6;17(22):5419. doi: 10.3390/ma17225419 (PMC11595300; doi:10.3390/ma17225419)
Supplement: Supplementary file 1 [file materials-17-05419-s001.zip › materials-3258401-supplementary.pdf]

Supplementary Materials

Analysis of Mechanical Properties and Thermal Conductivity of Thin-Ply Laminates in Ambient and Cryogenic Conditions

Anna Krzak [1,] *, Agnieszka J Nowak [1], Jirí Frolec [2], Tomáš Králík [2], Maciej Kotyk [3], Dariusz Boroński [3] and Grzegorz Matula [1]

Microscopic images of the cross-section surfaces of the tested laminates (Figures S1–S4) were captured using a Leica DVM6 digital microscope (from Leica Microsystems, offering precise 2D and 3D image analysis) at 30x magnification.

Analysing the microscopic images, it can be concluded that all materials exhibited discontinuities in their structure at room temperature. No signs of structural delamination, circular indentations, or air bubbles were observed. This indicates proper resin saturation and high technological quality, enabling adequate impregnation of the substrate, which is evidence of successful production. Some of the tested composite materials displayed partial structural failure under cryogenic conditions, while only in the case of EP_1_1 was characteristic delamination observed. Weaker mechanical and thermal properties characterize this material.

Figures S5–S8 present microphotographs of the surface of composite materials at 500x magnification. These microphotographs were taken using the FIB-SEM Helios scanning electron microscopeequipped with Everhart-Thornley detector at room temperature (RT) before and after treatment of the samples in liquid Helium (LHe). Based on the observations, it appears that the exposure had no effect. Only in the case of EP_2_2 were voids observed, which may have been caused by bubbles during the manufacturing of the sample material.

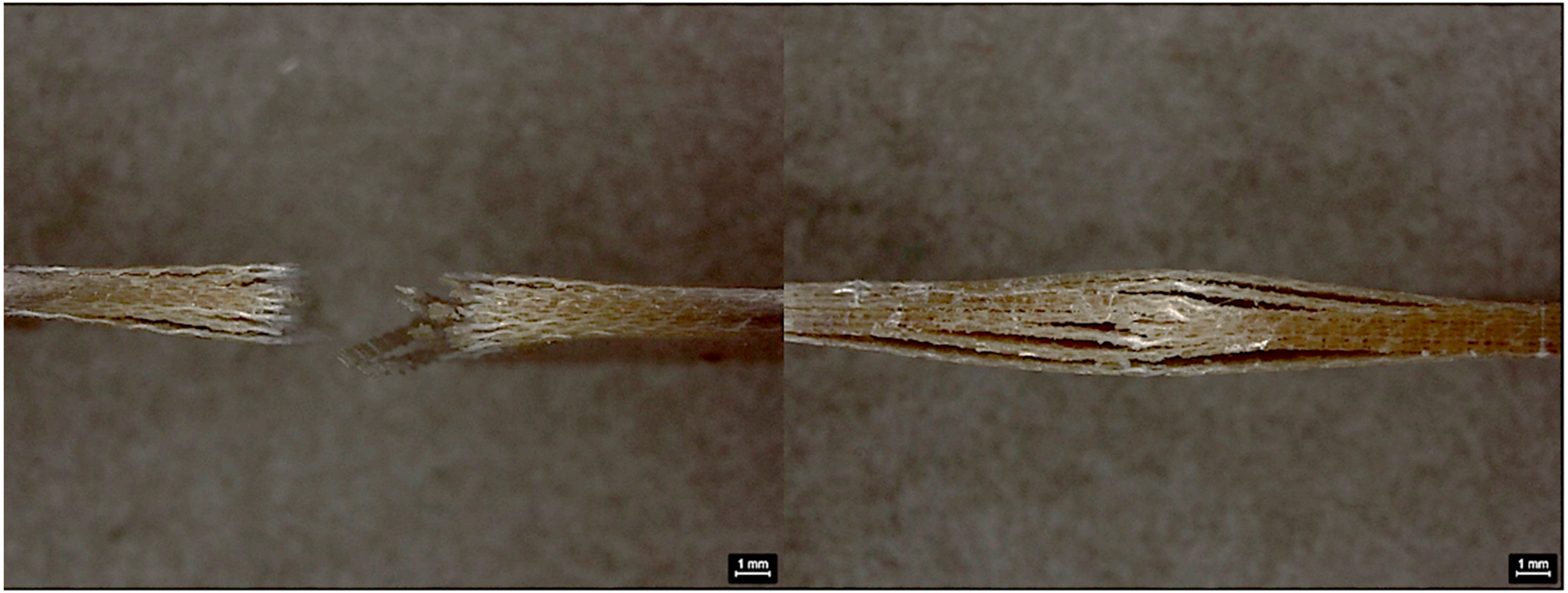

Figure S1. Microscope images of EP_1_1 laminates in 30x magnification, tested in RT (left) and in LN (right).

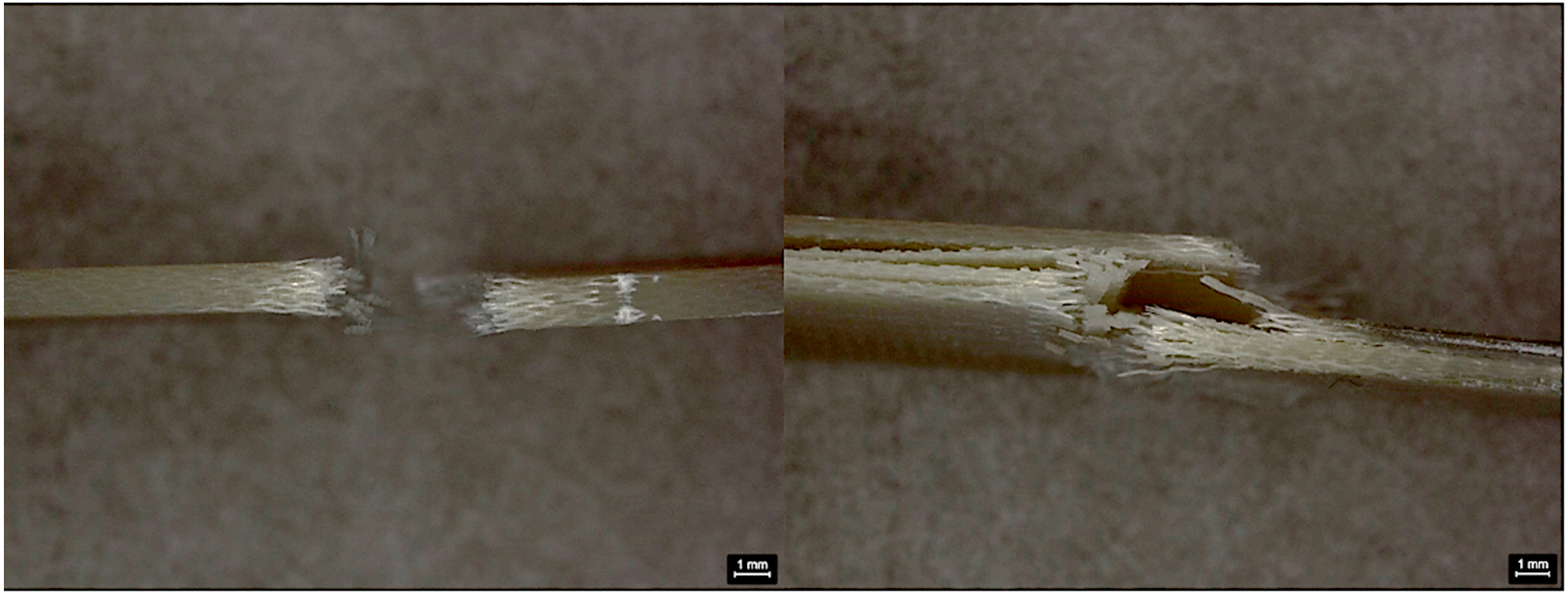

Figure S2. Microscope images of EP_1_3 laminates in 30x magnification, tested in RT (left) and in LN (right).

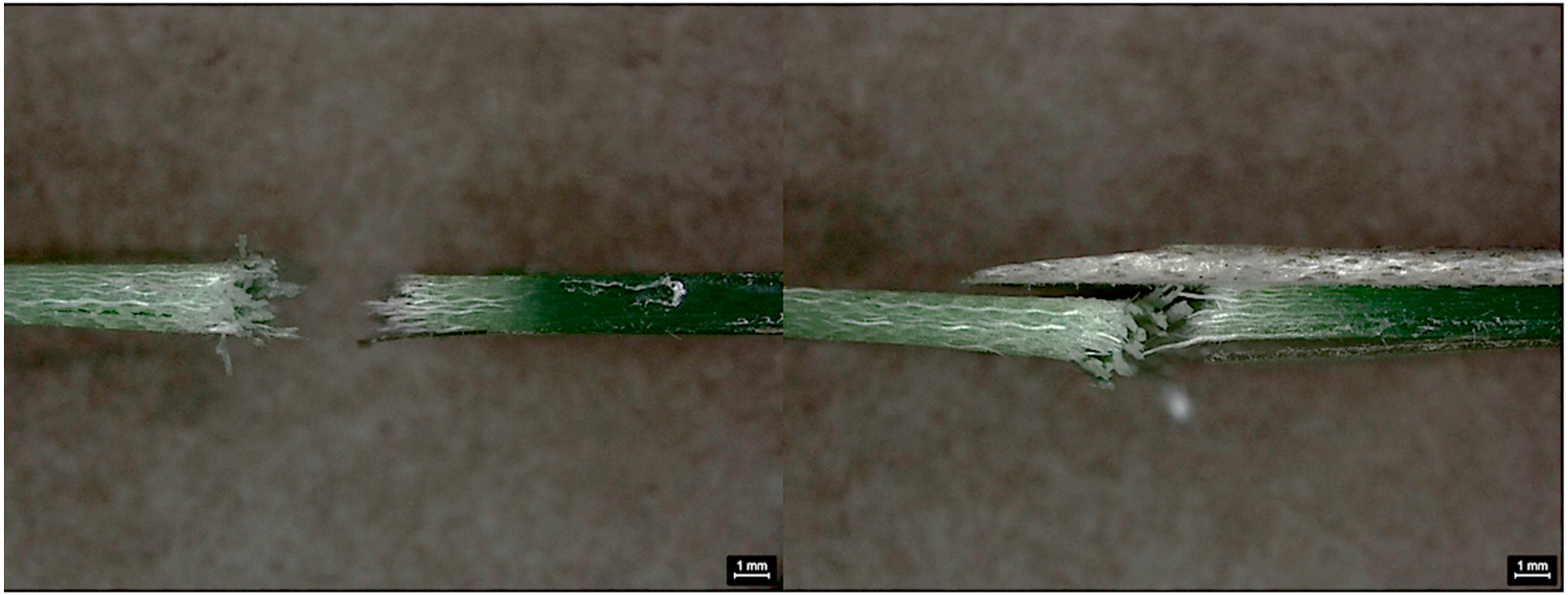

Figure S3. Microscope images of EP_2_1 laminates in 30x magnification, tested in RT (left) and in LN (right).

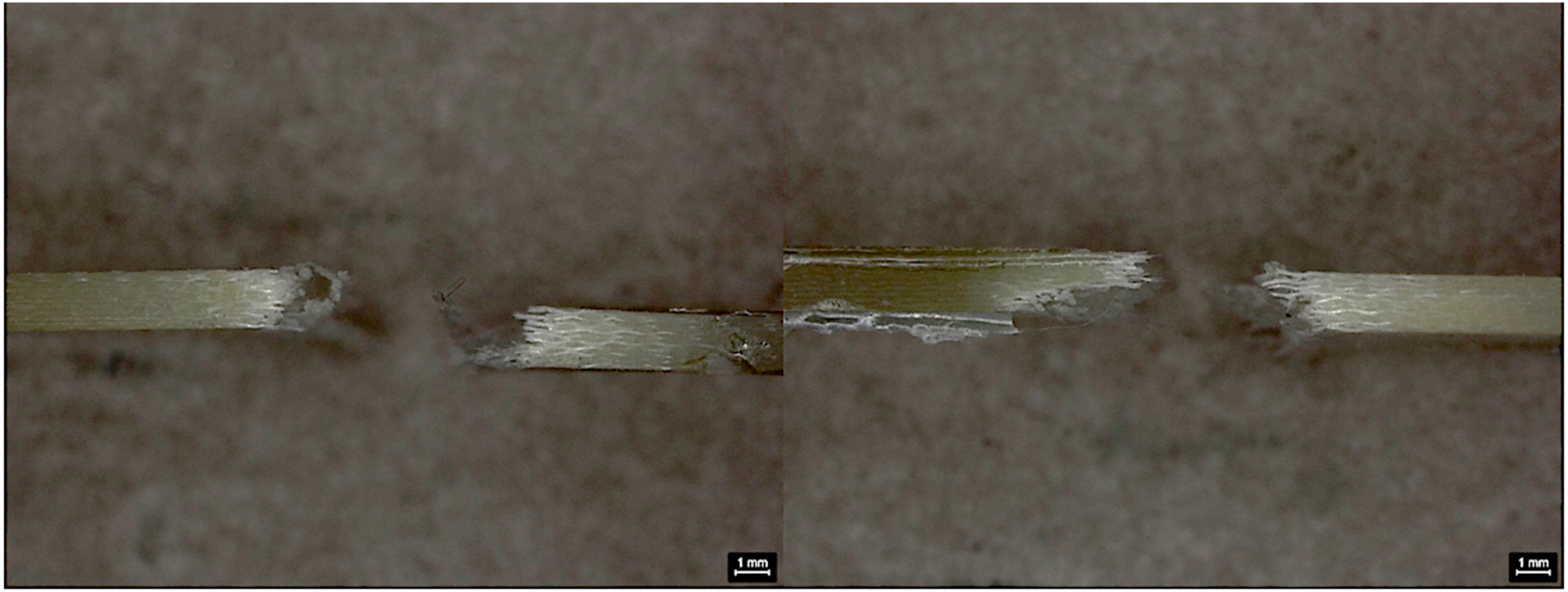

Figure S4. Microscope images of EP_2_2 laminates in 30x magnification, tested in RT (left) and in LN (right).

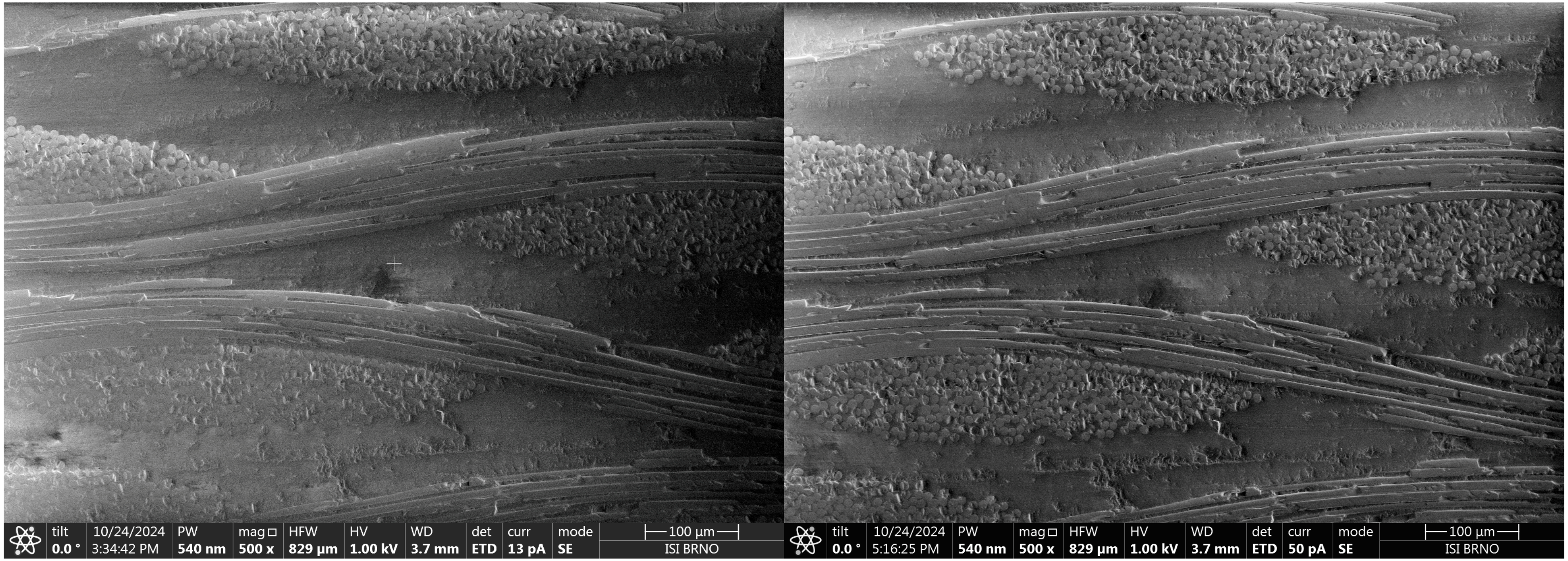

Figure S5. SEM image of EP_1_1 laminate at 500x magnification taken at RT before (left) and after (right) immersion of the sample in LHe.

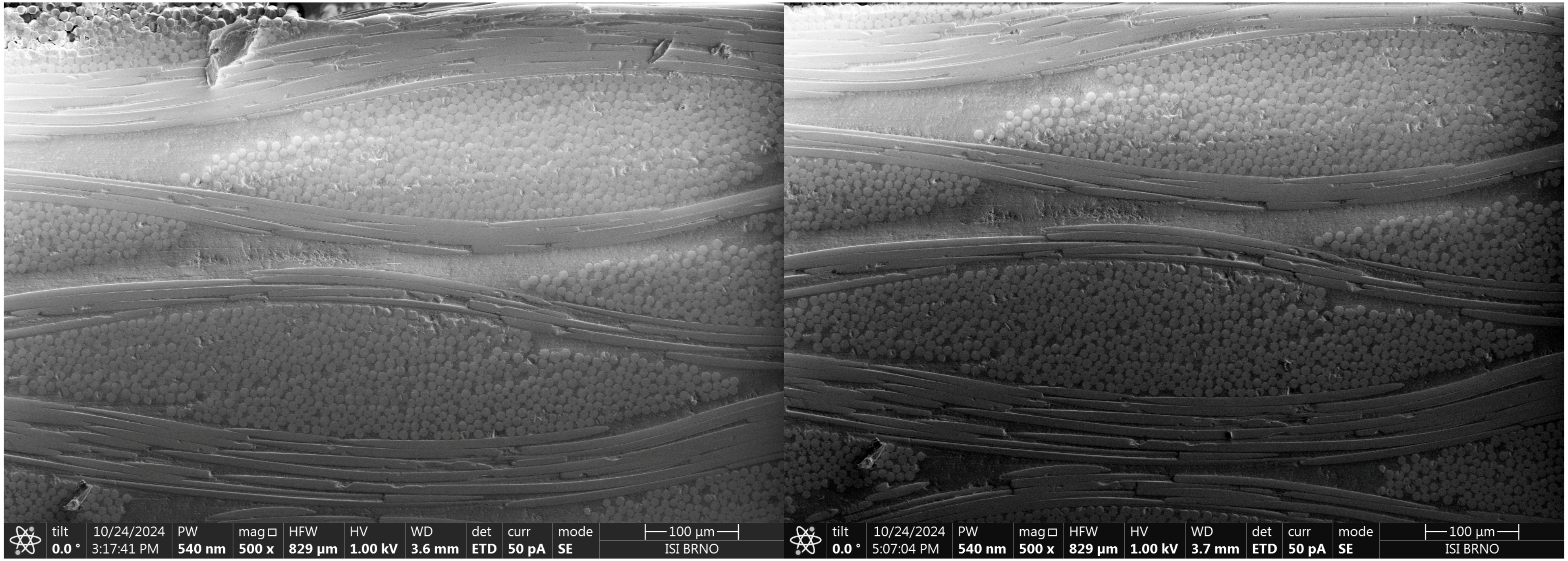

Figure S6. SEM image of EP_1_3 laminate at 500x magnification, taken at RT before (left) and after (right) immersion of the sample in LHe.

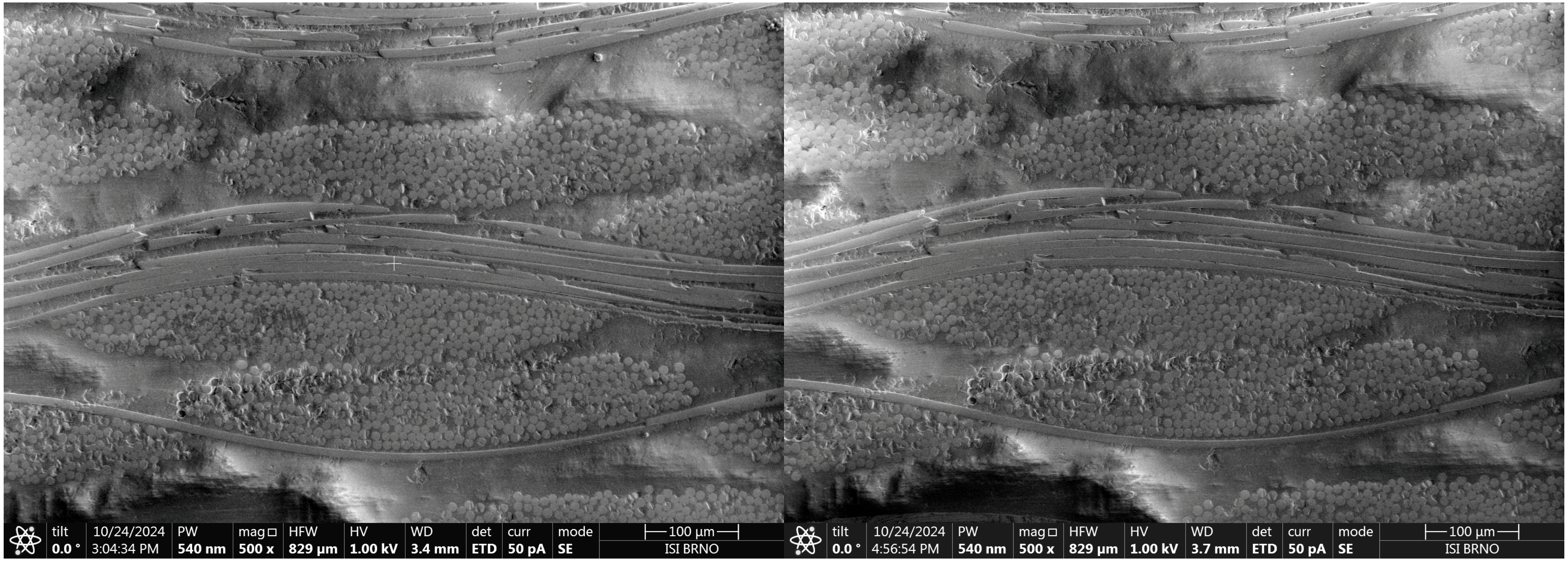

Figure S7. SEM image of EP_2_1 laminate at 500x magnification, taken at RT before (left) and after (right) immersion of the sample in LHe.

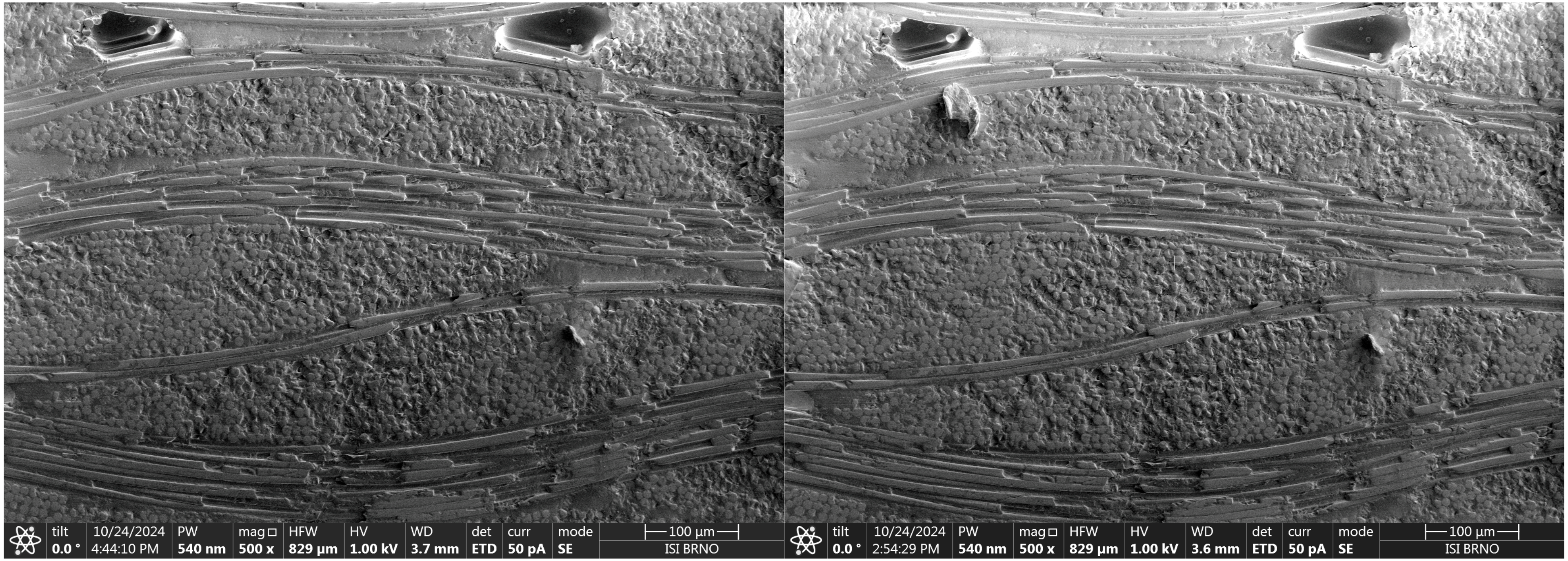

Figure S8. SEM image of EP_2_2 laminate at 500x magnification, taken at RT before (left) and after (right) immersion of the sample in LHe.
